# Inhibition of the gyrA promoter by transcription-coupled DNA supercoiling in *Escherichia coli*

**DOI:** 10.1038/s41598-018-33089-4

**Published:** 2018-10-03

**Authors:** Samantha Dages, Kelley Dages, Xiaoduo Zhi, Fenfei Leng

**Affiliations:** 1 Biomolecular Sciences Institute, Florida International University, 11200 SW 8th Street, Miami, FL 33199 USA; 2 Department of Chemistry & Biochemistry, Florida International University, 11200 SW 8th Street, Miami, FL 33199 USA

## Abstract

The *E. coli* gyrA promoter (P_gyrA_) is a DNA supercoiling sensitive promoter, stimulated by relaxation of DNA templates, and inhibited by (−) DNA supercoiling in bacteria. However, whether P_gyrA_ can be inhibited by transient and localized transcription-coupled DNA supercoiling (TCDS) has not been fully examined. In this paper, using different DNA templates including the *E. coli* chromosome, we show that transient and localized TCDS strongly inhibits P_gyrA_ in *E. coli*. This result can be explained by a twin-supercoiled domain model of transcription in which (+) and (−) supercoiled domains are generated around the transcribing RNA polymerase. We also find that fluoroquinolones, such as ciprofloxacin, can substantially increase the expression of the firefly luciferase under the control of the P_gyrA_ coupled to a divergent IPTG-inducible promoter in the presence of IPTG. This stimulation of P_gyrA_ by fluoroquinolones can be also explained by the twin-supercoiled domain model of transcription. This unique property of TCDS may be configured into a high throughput-screening (HTS) assay to identify antimicrobial compounds targeting bacterial DNA gyrase.

## Introduction

DNA supercoiling plays a critical role in several crucial DNA transactions including DNA replication, recombination, transcription, and DNA repair^1,2^. In bacteria, DNA molecules are usually (−) supercoiled^3^. DNA supercoiling *in vivo* is determined by counteractions of DNA topoisomerase I & IV (relaxation) and DNA gyrase ((−) supercoiling)^4,5^. Inhibition of DNA gyrase activities by gyrase inhibitors causes the relaxation of the DNA templates or accumulation of (+) supercoiled plasmids^6^ and also induces the expression of DNA gyrase in bacteria^7^. Deletion of *topA* from the chromosome results in the production of hypernegatively supercoiled DNA molecules at the exponential phase of bacteria^8^. Recent genomic studies also showed that DNA supercoiling is critical for transcription regulation of many genes during bacterial cell growth^9–13^.

Transcription can also disrupt localized DNA supercoiling *in vitro*^14–18^ and *in vivo*^6,8,19–23^. Liu and Wang formulated a twin supercoiled domain model of transcription to explain how transcription affects localized DNA supercoiling^24^. As the length of the RNA transcript increases, it becomes more and more difficult for the RNA-RNA polymerase complex to rotate around the DNA molecule. At a turning point, energetically, it is more practical to rotate the DNA about its own helical axis. Further translocation of the RNA-RNA polymerase along the DNA template generates a positively supercoiled domain in front of the transcribing RNA polymerase and a negatively supercoiled domain behind it^24^. Many *in vitro* and *in vivo* results support this twin supercoiled domain model. For instance, in defined protein systems, transcription is able to drive close circular DNA templates to hypernegatively supercoiled status in the presence of DNA gyrase because DNA gyrase converts a fraction of the transient (+) supercoils into permanent (−) supercoils^14–18^. Likewise, in *E. coli topA* strains, transcription at the exponential phase is able to drive close circular DNA templates to hypernegatively supercoiled because DNA gyrase converts (+) supercoiled domain into (−) supercoils^6,8,19–23^.

Transcription-coupled DNA supercoiling (TCDS) is also able to activate supercoiling-sensitive promoters in bacteria^25–29^. The best-studied case is the activation of bacterial Leu-500 promoter (P_leu-500_) by TCDS, a promoter containing a single A-to-G mutation in the promoter region of the *leu* operon^30,31^. Previous studies demonstrated that transcription-driven localized supercoiling rather than global supercoiling density was responsible for the activation of P_leu-500_^25–27,32–37^. The orientation of TCDS had opposite effects where (−) supercoiling domain activated P_leu-500_ and (+) supercoiling domain suppressed the promoter^27^. In our previously published studies^38^, using uniquely designed linear plasmids, we demonstrated that transient and localized (−) DNA supercoiling can strongly activate P_leu-500_. The activation of P_leu-500_ is dependent of the promoter strength and the length of RNA transcripts, unique properties of TCDS as predicted by the twin-supercoiled domain mechanism. We also demonstrated that TCDS could be generated on topologically open DNA molecules *in E. coli* cells. These results suggest that topological boundaries or barriers are not necessary for the generation of TCDS *in vivo*.

The *E. coli gyrA* promoter (P_gyrA_) is another supercoiling sensitive promoter and stimulated by relaxation of DNA templates^7,39,40^. Early mutation studies showed that the stimulation of P_gyrA_ stems from a 20 bp DNA sequence around the −10 region of P_gyrA_^39,40^. Since this 20 bp DNA sequence is intrinsically bent or curved^41^, it is possible that the DNA bend or curvature functions as a supercoiling sensor for the activation by DNA relaxation^41^. Nevertheless, whether P_gyrA_ can be inhibited by TCDS has not been examined. Here, using different DNA templates including the *E. coli* chromosome, we show that transient and localized (−) TCDS is able to strongly inhibit P_gyrA_ in *E. coli*. We also found that fluoroquinolones, such as ciprofloxacin, were able to substantially increase the expression of the firefly luciferase controlled by P_gyrA_ coupled to a divergent IPTG-inducible promoter in the presence of IPTG. This unique property of TCDS may be used to screen and identify antimicrobial compounds targeting bacterial DNA gyrase.

## Results and Discussion

**Figure 1:**
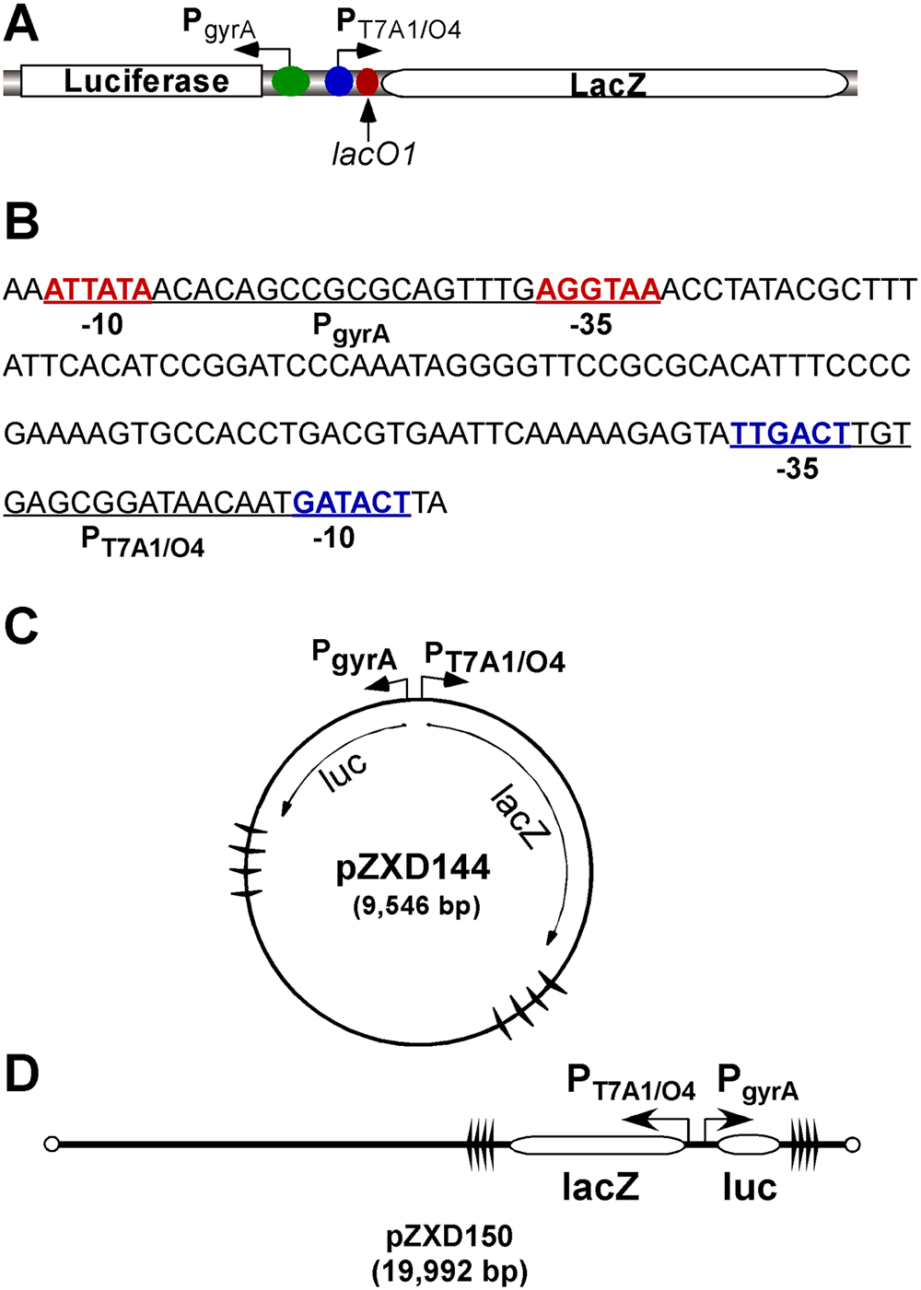
Experimental design of a pair of divergently coupled transcription units to examine transcription inhibition of P_gyrA_ by TCDS *in vivo*. (**A**) Divergently coupled promoters P_T7A1/O4_ and P_gyrA_, respectively, control the expression of β-galactosidase (*lacZ*) and firefly luciferase (*luc*). (**B**) The DNA sequence of the pair of divergently coupled promoters, P_T7A1/O4_ and P_gyrA_. Underlined are P_gyrA_ and P_T7A1/O4_ with −10 and −35 regions. (**C**,**D**) Maps of circular plasmid pZXD144 and linear plasmid pZXD150. Winged triangles represent Rho-independent *rrnB* T1 transcription terminators.

**Figure 2:**
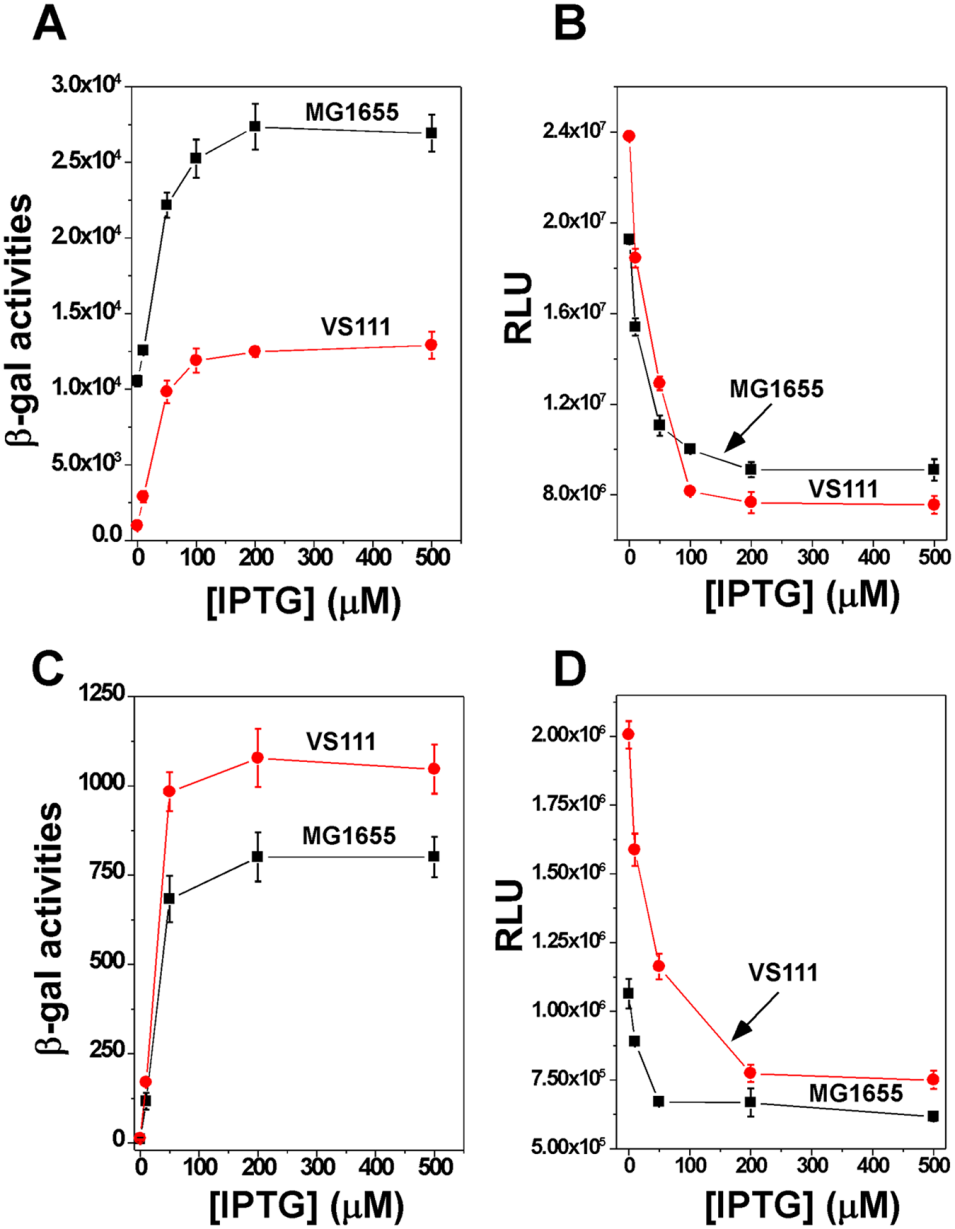
Inhibition of P_gyrA_ by TCDS for circular plasmid pZXD144 (**A**,**B**) and linear plasmid pZXD150 (**C,** **D**). The activities of β-galactosidase (Miller’s units) and firefly luciferase (RLU, relative light units) were determined as described under Methods and plotted versus the IPTG concentration. (**A**,**B**) *E. coli* strains *MG1655(DE3)ΔlacZ* (black squares and lines) and *VS111(DE3)ΔlacZ* (red circles and lines) carrying pZXD144 were used. (**C**,**D**) *E. coli* strains *MG1655(DE3)ΔlacZ* (black squares and lines) and *VS111(DE3)ΔlacZ* (red circles and lines) carrying pZXD150 were used. The standard deviation (SD) was determined according to results from three independent experiments.

In our previous studies^38^, using an *in vivo* system that contains *E. coli topA* strain *VS111(DE3)ΔlacZ* or wild-type strain *MG1655(DE3)ΔlacZ* and a circular or linear plasmid DNA template, we demonstrated that transient and localized TCDS from a divergently-coupled transcription unit potently activated the supercoiling-sensitive promoter P_leu-500_. In this study, we decided to utilize this system to examine whether and how TCDS inhibits a different supercoiling-sensitive promoter P_gyrA_. For this purpose, we substituted P_leu-500_ with P_gyrA_ divergently coupled to the strong IPTG-inducible promoter P_T7A1/O4_ (Fig. 1). The distance between these two promoters is 92 bp (Fig. 1A). As shown in Fig. 1C,D, we used 2 sets of 4 Rho-independent, *rrnB T1* transcription terminators to block transcription from P_T7A1/O4_ and P_gyrA_, respectively. In this case, transcription is restricted to a selected region of the plasmids^22^. Circular plasmid pZXD144 and linear plasmid pZXD150 were used to transform *VS111(DE3)ΔlacZ* or *MG1655(DE3)ΔlacZ*. After IPTG was added to *E. coli* cells in the early log phase, luciferase activities were used to monitor the inhibition of P_gyrA_. Results in Fig. 2 show that TCDS strongly inhibits the supercoiling-sensitive P_gyrA_ for both circular and linear plasmids. For example, TCDS from *E. coli* RNA polymerase on pZXD144 inhibited 53% and 68% of P_gyrA_ in *VS111(DE3)ΔlacZ* and *MG1655(DE3)ΔlacZ*, respectively, comparing with the activities of P_gyrA_ in the absence of IPTG (Fig. 2B). TCDS on pZXD150 inhibited 42% and 63% of P_gyrA_ in *VS111(DE3)ΔlacZ* and *MG1655(DE3)ΔlacZ*, respectively (Fig. 2D). Due to the fact that linear DNA templates cannot be permanently supercoiled^42^, these results unambiguously demonstrated that transient and localized TCDS, rather than global supercoiling, inhibits the divergently coupled P_gyrA_. Interestingly, for circular plasmid pZXD144, the expression level of β-galactosidase is always higher in *MG1655(DE3)ΔlacZ* than that in *VS111(DE3)ΔlacZ* in the absence or presence of IPTG (Fig. 2A), which is consistent with our previously published results^43^. In contrast, for linear plasmid pZXD150, the expression level of β-galactosidase is lower in *MG1655(DE3)ΔlacZ* comparing with that in *VS111(DE3)ΔlacZ* (Fig. 2C). These results suggest that DNA supercoiling plays some roles in regulating the activities of P_T7A1/O4_^38^. Please note that each *E. coli* cell carries approximate 1 copy of a linear plasmid, the overall expression levels of firefly luciferase are much lower for linear plasmids^38^. Since the *topA* strain *VS111* is a DNA topoisomerase I deletion strain, it should have greater supercoiling fluctuations when disturbed by TCDS. As a result, P_gyrA_ should be more sensitive to TCDS. Indeed, our results showed that P_gyrA_ is more sensitive to the IPTG concentration, indicating that it is more sensitive to TCDS (Fig. 2B and D).

**Figure 3:**
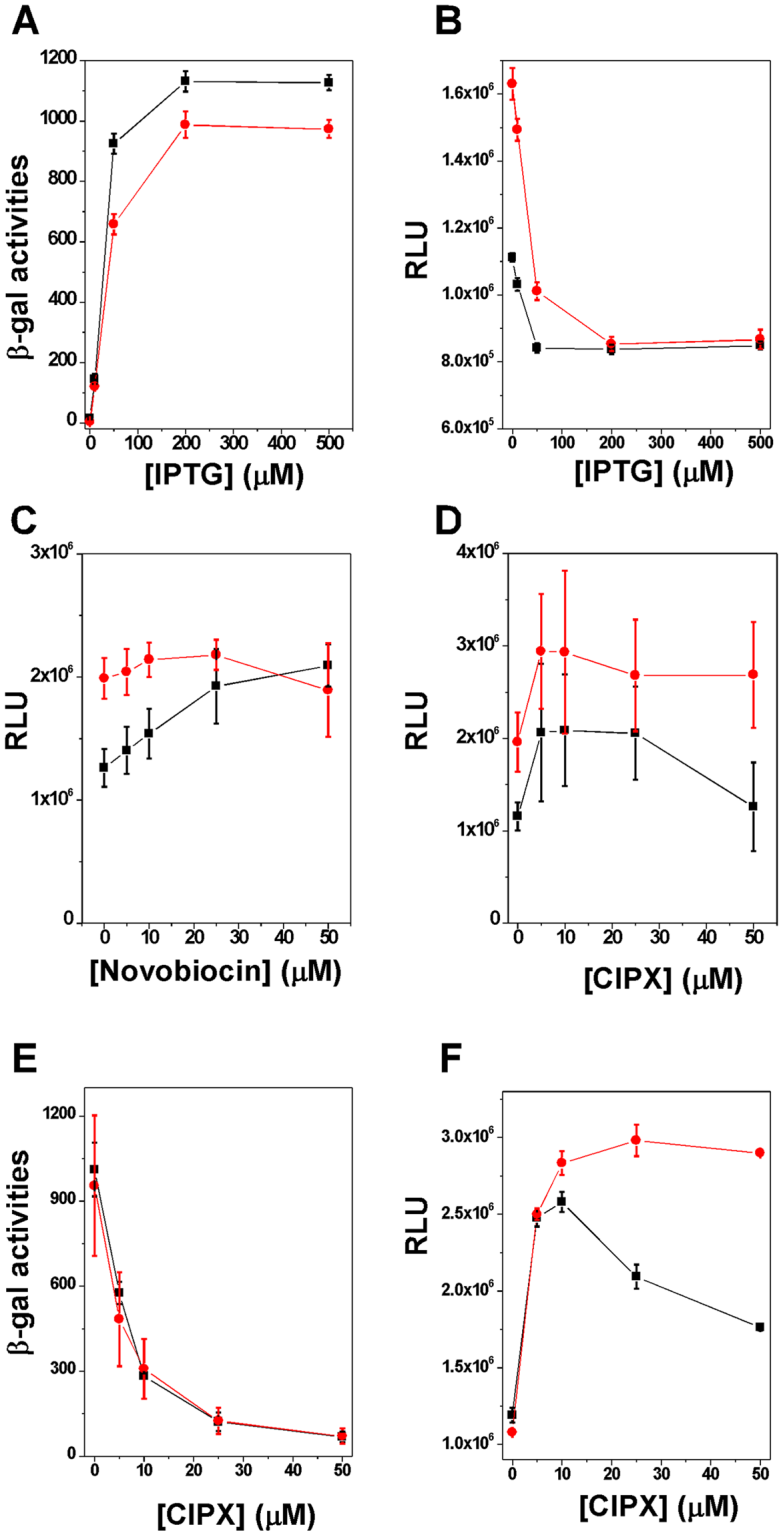
Strong inhibition of the supercoiling-sensitive P_gyrA_ by TCDS on the chromosome. (**A**,**B**) TCDS assays for P_gyrA_ on the chromosome. *E. coli* strains *FL1181* (*MG1655(DE3)ΔlacZ attTn7::P*_*T7A1/O4*_*lacZ-P*_*gyrA*_*luc*; black squares and lines) and *FL1182* (*VS111(DE3)ΔlacZ attTn7::P*_*T7A1/O4*_*lacZ-P*_*gyrA*_*luc*; red circles and lines) were used. The activities of β-galactosidase and firefly luciferase were determined as described under Methods and plotted versus the IPTG concentration. (**C**,**D**) Effects of novobiocin (**C**) and ciprofloxacin (**D**) on P_gyrA_ of *FL1181* (black squares and lines) and *FL1182* (red circles and lines) in the absence of IPTG. (**E**,**F**) DNA gyrase inhibitors significantly enhanced the expression of firefly luciferase for *FL1181* and *FL1182* in the presence of IPTG. Overnight cell cultures were diluted 100-fold and grown until OD600 reached ~0.2. Then 0.5 mM of IPTG and various concentrations of ciprofloxacin or other antibiotics were added to the cell cultures. After 30 min incubation, the activities of β-galactosidase and firefly luciferase were determined described under Methods. (**C**) Ciprofloxacin (CIXP) inhibited the expression of β-galactosidase. (**D**) CIXP greatly enhanced the expression of firefly luciferase. The standard deviation (SD) was determined according to results from three independent experiments.

Next, we examined how TCDS inhibits P_gyrA_ on the *E. coli* chromosome. First, we placed a ~5 kb DNA fragment carrying the divergently coupled P_gyrA_ and P_T7A1/O4_ promoters (Fig. 1A) into the *attTn7* site of the *E. coli* chromosome (Fig. S1; the 84.2 min of the *E. coli* chromosome^44^) using a procedure of transposon Tn7^45^ to yield a wild type strain *FL1181* (*MG1655(DE3)ΔlacZ attTn7::P*_*T7A1/O4*_*lacZ-P*_*gyrA*_*luc*) and a *topA* strain *FL1182* (*VS111(DE3)ΔlacZ attTn7::P*_*T7A1/O4*_*lacZ-P*_*gyrA*_*luc*). Due to technical difficulties, the four T1 transcription terminators were not included in these constructs. Similar to results for plasmid DNA templates as shown above, transcription by *E. coli* RNA polymerase can substantially inhibit transcription from P_gyrA_ on the *E. coli* chromosome (Fig. 3A,B). For example, TCDS was able to inhibit 24% and 47% of P_gyrA_ in *FL1181* and *FL1182*, respectively. Interestingly, in the absence of IPTG, P_gyrA_ in *FL1182* is more active than that in *FL1181* (Fig. 3B). As demonstrated previously^43^, in the absence of IPTG, P_T7A1/O4_ is much more active in the wildtype strain *MG1655* that that in the *topA* strain *VS111*. Although the DNA templates may be more negatively supercoiled globally in *VS111*, the localized supercoiling around P_gyrA_ in the wildtype strain *MG1655* should be more negatively supercoiled than that in *VS111* due to TCDS. In this way, the expression level of luciferase in *VS111* should be higher than that in *MG1655* in the absence of IPTG.

**Figure 4:**
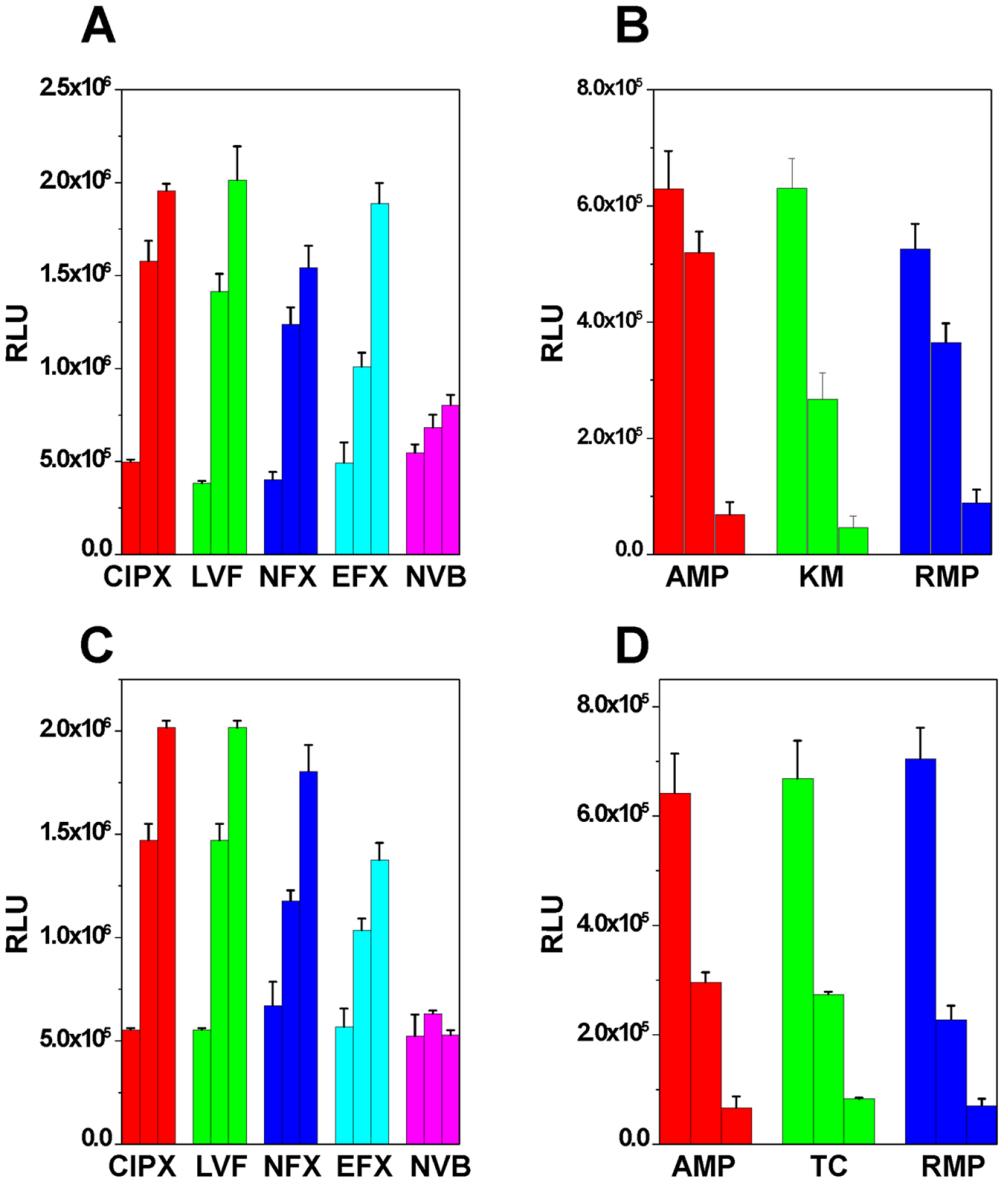
The stimulation of expression of firefly luciferase of *FL1181* (**A**) and *FL1182* (**C**) by fluoroquinolones in the presence of 0.5 mM IPTG. CIXP, LVF, EFX, NFX, and novobiocin represent ciprofloxacin, levofloxacin, enrofloxacin, norfloxacin, and novobiocin, respectively. Three bars from left to right represent luciferase activities in the presence of 0, 5, and 10 μM of fluoroquinolones, respectively. (**B** and **D**) The inhibition of expression of firefly luciferase by other antibiotics (none gyrase inhibitors) for *FL1181* (**B**) and *FL1182* (**D**). RMP, KM, AMP, and TC represent rifampicin, kanamycin, ampicillin, and tetracycline, respectively. The following are concentrations used in the experiments from left to right: AMP, 0, 150, 300 μM; KM, 0, 40, 80 μM; RMP, 0, 25, 50 μM; TC, 0, 10, 20 μM. The standard deviation (SD) was determined according to results from three independent experiments.

Since it was shown that gyrase inhibitors, such as coumermycin, quinolones, and novobiocin, are able to induce the expression of gyrA and gyrB in bacteria^46,47^, we also treated *FL1181* and *FL1182* with two gyrase inhibitors, novobiocin and ciprofloxacin, and examined whether these two gyrase inhibitors are able to increase the firefly luciferase expression under the control of P_gyrA_. At the early exponential stage, novobiocin slightly enhanced the expression of firefly luciferase in *FL1181* (Fig. 3C) and did not have much effect on the expression of firefly luciferase in the *topA* strain *FL1182* (Fig. 3C). Ciprofloxacin at low concentrations slightly stimulated the expression of firefly luciferase for both strains (Fig. 3D; the differences appear to be statistically insignificant) and inhibited the expression of firefly luciferase in *FL1181* at 50 μM (Fig. 3D). Intriguingly, in the presence of IPTG, the stimulation of firefly luciferase expression by ciprofloxacin was significantly amplified (Fig. 3F) although ciprofloxacin at high concentrations completely inhibited the expression of β-galactosidase for both strains (Fig. 3E). We noticed some differences between these two *E. coli* strains. For the wild type strain *FL1181*, the stimulation of firefly luciferase expression by ciprofloxacin decreased at higher concentrations, i.e., 20 and 50 μM. For *topA* strain *FL1182*, however, the stimulation by ciprofloxacin plateaued at 10 μM and stayed high at 50 μM. These results suggest that topoisomerase I plays a role in the regulation of P_gyrA_ activities in *E. coli*. We further tested several other gyrase inhibitors including levofloxacin, norfloxacin, enrofloxacin, and novobiocin, and found that only fluoroquinolones dramatically stimulated the expression of firefly luciferase in *FL1181* and *FL1182* in the presence of IPTG (Fig. 4A and C). Novobiocin’s effect on the expression of firefly luciferase is negligible for both strains (Fig. 4A and C). At the tested concentrations, i.e., 5 and 10 µM, these fluoroquinolones slightly inhibit the growth of the two *E. coli* strains (Fig. S2). We also tested several other types of antibiotics, such as transcription inhibitors (rifampicin), protein synthesis inhibitors (kanamycin and tetracycline), and cell wall synthesis inhibitors (ampicillin), and found that all these antibiotics inhibited the expression of firefly luciferase in *FL1181* and *FL1182* (Figs 4B,D and S3). These results suggest that the enhancement of the expression of firefly luciferase is specific for gyrase inhibitors, especially for fluoroquinolones. These results also suggest that this stimulation assay can be used to identify antibiotics targeting bacterial DNA gyrase.

We believe that the twin supercoiled domain model of transcription^24^ can explain why gyrase inhibitors are able to stimulate the expression of firefly luciferase in *FL1181* and *FL1182*. At the early exponential phase, RNA polymerase is actively transcribing genes along the *E. coli* chromosome, introducing localized DNA supercoiling around these genes, and remodeling the chromosome. For *FL1181* and *FL1182*, the divergently coupled P_gyrA_ and P_T7A1/O4_ promoters with the *luc* and *lacZ* genes are located at 84.2 min of the *E. coli* chromosome near the seven rRNA operons^48^. Since the *E. coli* RNA polymerase transcribes along these seven rRNA operons away from 84.2 min of the *E. coli* chromosome, transcription should introduce significant amounts of (−) supercoils to this region. As a result, P_gyrA_ is repressed. For the wild type strain *FL1181*, in the presence of novobiocin, DNA gyrase is no longer capable of removing (+) supercoiled domain generated during transcription. Topoisomerase I, on the other hand, relaxes (−) supercoiled domain. In this way, DNA templates including the chromosome should be more relaxed, which resulted in the stimulation of the expression of firefly luciferase under the control of P_gyrA_ (Fig. 3C). Since the *topA* strain *FL1182* does not have DNA topoisomerase I to remove (−) supercoiled domain, the DNA supercoiling status in *FL1182* will not fluctuate significantly in the presence of novobiocin. This is the reason why novobiocin did not greatly affect the expression of firefly luciferase in *FL1182* (Fig. 3C). Ciprofloxacin is a different DNA gyrase inhibitor and forms gyrase-cipro-DNA complexes that cause the termination of transcription for both *FL1181* and *FL1182* (Fig. 3E). The (−) supercoiled domain should not be formed. As a result, ciprofloxacin was able to “stimulate” the expression of firefly luciferase for both strains (Fig. 3D).

**Figure 5:**
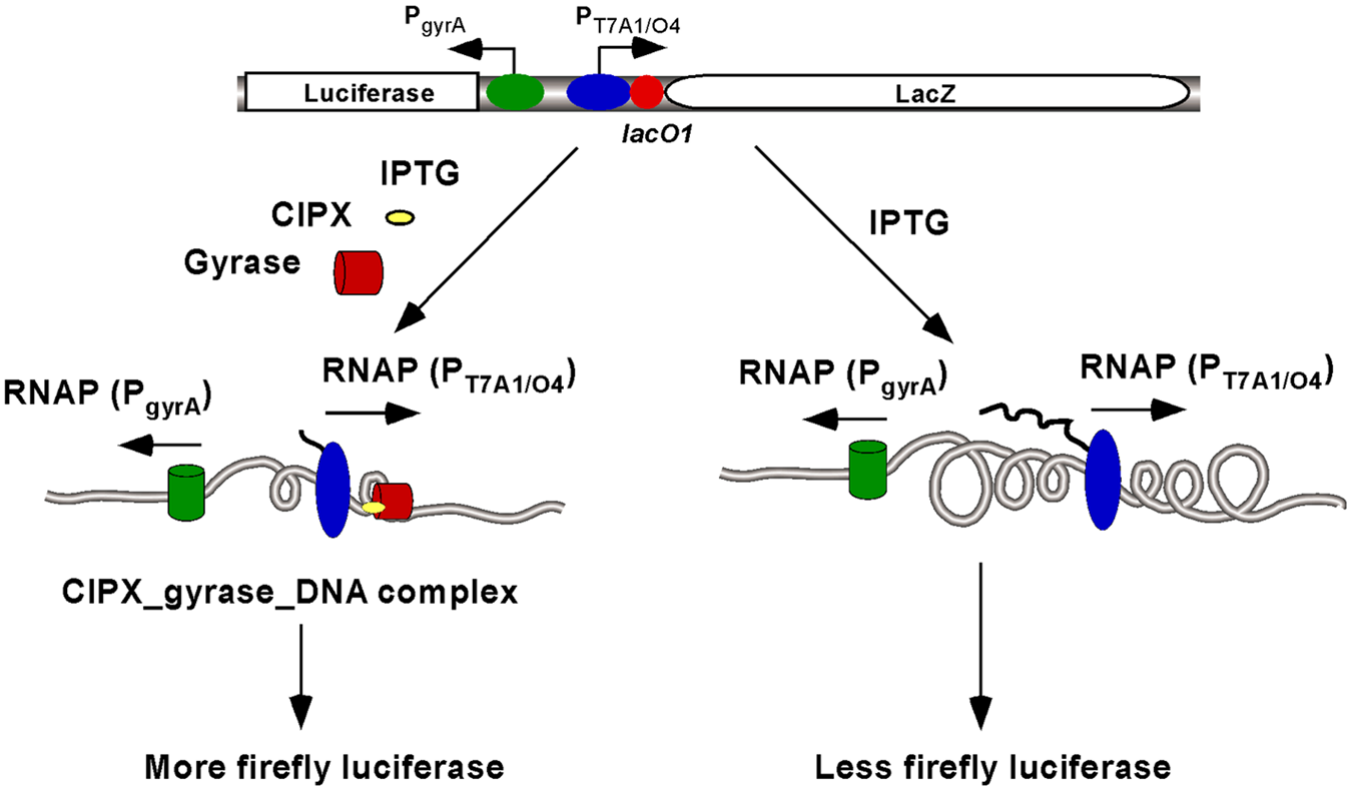
A possible mechanism to explain effects of ciprofloxacin on P_gyrA_ in the presence of IPTG. In the presence of IPTG (right panel), transcription from P_T7A1/O4_ induces significant TCDS and inhibits the expression of firefly luciferase from P_gyrA_. However, in the presence of gyrase inhibitor ciprofloxacin, ciprofloxacin stabilizes gyrase-cipro-DNA complex that blocks transcription from P_T7A1/O4_. The (−) supercoils behind RNA polymerase are not formed. As a result, the expression of firefly luciferase is “enhanced.”

Regarding why fluoroquinolones, in the presence of IPTG, are able to enhance the expression of firefly luciferase (Fig. 4A and C), we favor the model depicted in Fig. 5 for explanation. In the presence of IPTG, transcription initiated from the strong P_T7A1/O4_ produces a significant amount of (−) supercoils behind the RNA polymerase and as a result inhibits the expression of firefly luciferase by P_gyrA_ (Fig. 3B). However, ciprofloxacin stabilizes gyrase-cipro-DNA complexes for those DNA gyrases that remove the (+) supercoiled domain in front of RNA polymerase. As a result, transcription from P_T7A1/O4_ is terminated (Fig. 3E) and the (−) supercoiling domain behind the RNA polymerase is not formed. Because P_gyrA_ is a weak promoter and transcription from P_gyrA_ should not produce significant amounts of (+) supercoils in front of RNA polymerase, gyrase-cipro-DNA complexes are not formed. In this scenario, ciprofloxacin will not be able to inhibit the expression of firefly luciferase. In contrast, the (−) DNA supercoiled domain from the divergently coupled P_T7A1/O4_ is not formed, the expression of firefly luciferase is greatly “enhanced” (Fig. 3F). Because novobiocin only inhibits DNA gyrase activities and does not form gyrase-novobiocin-DNA complexes, it should not significantly enhance or inhibit the expression of firefly luciferase in *FL1181* and *FL1182* (Fig. 4A and C). Other antibiotics, due to not affecting DNA supercoiling status *in vivo*, should not be able to enhance the expression of firefly luciferase. In contrast, they inhibited the expression of firefly luciferase and β-glactosidase in *FL1181* and *FL1182*.

### Summary

Here, using a unique *in vivo* system, we demonstrated that transient and localized (−) TCDS provided by *E. coli* RNA polymerase could inhibit the P_gyrA_ at the plasmid and chromosomal levels. We also found that fluoroquinolones, such as ciprofloxacin, were able to substantially increase the expression of the firefly luciferase under the control of the P_gyrA_ in the presence of IPTG. This unique property of TCDS can be effectively used to screen and identify antimicrobial compounds targeting bacterial DNA gyrase.

## Methods

### Materials

Kanamycin, lysozyme, and ortho-Nitrophenyl-β-galactoside (ONPG) were purchased from Sigma-Aldrich Corporation (St. Louis, MO). Ampicillin and bovine serum albumin (BSA) were bought from Fisher Scientific (Fairlawn, NJ). Isopropyl-β-D-thiogalactopyranoside (IPTG) was obtained from Anatrace, Inc (Maumee, Ohio). All restriction enzymes, T4 DNA ligase, and T4 polynucleotide kinase were purchased from New England Biolabs (Beverly, MA). *Pfu* DNA polymerase was obtained from Stratagene, Inc. (La Jolla, CA). All synthetic oligonucleotides were purchased from Eurofins Genomics (Huntsville, AL). Plasmid and DNA fragment cleaning kits including QIAprep Spin Miniprep Kit, QIAquick Gel Extraction Kit, and QIAquick Nucleotide Removal Kit were obtained from QIAGEN, Inc. (Valencia, CA). Luciferase Assay System was bought from Promega Corporation (Madison, WI).

### Plasmid DNA templates

Circular plasmid pZXD133, a derivative of pBR322, was described previously^43^. Plasmid pZXD144 was constructed by inserting a 70 bp synthetic oligomer harboring a P_gyrA_ into the BamHI and HindIII sites of pZXD133. In this case, P_gyrA_ is divergently coupled to P_T7A1/O4_ (Fig. 1). Linear plasmid pZXD150 was described previously^38^.

### Bacterial strains

*E. coli* strains *MG1655*(*DE3*) and *VS111*(*DE3*) were described previously^22,23,43^. *E. coli* strains *FL1181* (*MG1655*(*DE3*)*ΔlacZ attTn7*::*P*_*T7A1/O4*_*lacZ*-*P*_*gyrA*_*luc*) and *FL1182* (*VS111*(*DE3*)*ΔlacZ attTn7*::*P*_*T7A1/O4*_*lacZ*-*P*_*gyrA*_*luc*) were created by utilizing a Tn7-based site-specific recombination system^45^ as follows. A 5.1 kb DNA fragment harboring the divergently coupled P_gyrA_ and P_T7A1/O4_ promoters controlling the *luc* and *lacZ* genes, respectively, was inserted into the *attTn*7 site of the *E. coli* chromosome^44^ (84 min) to yield *FL1181* and *FL1182* in which the IPTG-inducible P_T7A1/O4_ controls the expression of β-galactosidase.

### The expression of β-galactosidase

The expression level of β-galactosidase was measured as described in previous publications^38,49^. Briefly, 100 mL of LB was inoculated with 1 mL of overnight bacterial cell culture at ratio of 1:100 until OD_600_ = ~0.2. 100 μL of bacterial cell culture was added to 900 μL of Z-buffer (60 mM Na_2_HPO_4_, 40 mM NaH_2_PO_4_, 10 mM KCl, 1 mM MgSO_4_, and 50 mM β-mercaptoethanol). Then, 60 μL of chloroform and 30 μL of 0.1% SDS were added to lyse the cells. After cell lysates were incubated at 30 °C for 5 minutes, 200 μL of ONPG (4 mg/mL) was added. After another 15 min of incubation at 30 °C, 500 μL of 1 M Na_2_CO_3_ was added to stop the reaction. After cell debris was removed by centrifugation at 13,000 rpm for 1 min, the OD_420_ and OD_550_ values were measured in a Cary 50 spectrophotometer. β-Galactosidase activities (E) were calculated using equation:

1 $${\boldsymbol{E}}=1000\times \frac{{\boldsymbol{O}}{{\boldsymbol{D}}}_{420}-1.75\times {\boldsymbol{O}}{{\boldsymbol{D}}}_{550}}{{\boldsymbol{t}}\times {\boldsymbol{v}}\times {\boldsymbol{O}}{{\boldsymbol{D}}}_{600}}$$

where *t* and *v*, respectively, represent reaction time and cell culture volume.

### Luciferase assay

The expression of the firefly luciferase in *E. coli* were monitored by using the luciferase assay as described in our previous publication^38^.

## Electronic supplementary material

Supplementary Information
