# Supplementary material for: Inhibition of the gyrA promoter by transcription-coupled DNA supercoiling in Escherichia coli
Source: Sci Rep. 2018 Oct 3;8:14759. doi: 10.1038/s41598-018-33089-4 (PMC6170449; doi:10.1038/s41598-018-33089-4)
Supplement: Supplementary file 1 — Supplementary Information [file 41598_2018_33089_MOESM1_ESM.docx]

**Supplementary Information**

**Inhibition of the *gyrA* promoter by transcription-coupled DNA supercoiling in *Escherichia coli***

Samantha Dages^1,2^, Kelley Dages^1,2^, Xiaoduo Zhi^1,2^, and Fenfei Leng^1,2^*

From Biomolecular Sciences Institute^1^ and Department of Chemistry & Biochemistry^2^, Florida International University, 11200 SW 8^th^ Street, Miami, FL 33199

Running title: Inhibition of the *gyrA* promoter by TCDS

To whom correspondence should be addressed: Prof. Fenfei Leng, Department of Chemistry & Biochemistry, Florida International University, 11200 SW 8^th^ Street, Miami, FL 33199. Telephone: (305) 348-3277; Fax: (305) 348-3772; E-mail: [lengf@fiu.edu](mailto:lengf@fiu.edu)

**Figure S1.** The 100 minute map of the *E. coli* genome. The DNA replication origin (Oric), the termination site (terC), and the the *attTn7* site are shown.

**Figure S2.** **Inhibition of cell growth by different antibiotics.** Overnight cell cultures were diluted 100-fold and grown until OD_600_ reached 0.2. Then 0.5 mM of IPTG and various concentrations of antibiotics were added to the cell cultures. After additional 30 min incubation, OD600 was measured. (A) & (B) E. coli strain FL1181. (C) and (D) *E. coli* strain FL1182. CIPX, LVF, NFX, EFX, and NVB represent ciprofloxacin, levofloxacin, norfloxacin, enrofloxacin, and novobiocin, respectively. Three bars from left to right represent luciferase activities in the presence of 0, 5, and 10 μM of fluoroquinolones, respectively. AMP, KM, RMP, and TC represent ampicillin, kanamycin, rifampicin, and tetracycline, respectively. The following are concentrations used in the experiments from left to right: AMP, 0, 150, 300 μM; KM, 0, 40, 80 μM; RMP, 0, 25, 50 μM; TC, 0, 10, 20 μM. The standard deviation was calculated according to three independent experiments.

**Figure S3. Inhibition of the expression of b-galactosidase by ampicillin (AMP), tetracycline (TC), and rifampicin (RMP) for E. coli strain FL1181.** Overnight cell cultures were diluted 100-fold and grown until OD_600_ reached 0.2. Then 0.5 mM of IPTG and various concentrations of antibiotics were added to the cell cultures. After additional 30 min incubation, the activities of β-galactosidase were measured as described under Materials and Methods. The following are concentrations used in the experiments from left to right: AMP, 0, 150, 300 μM; RMP, 0, 25, 50 μM; TC, 0, 10, 20 μM. The standard deviation was calculated according to three independent experiments.
